# Preschool children's social skills, problem behaviors, academic self-esteem and teacher-child relationship: a serial mediation model

**DOI:** 10.3389/fpsyg.2025.1453193

**Published:** 2025-03-17

**Authors:** Cansu Tutkun, Seda Eskidemir Meral

**Affiliations:** ^1^Department of Preschool Education, Faculty of Education, Bayburt University, Bayburt, Türkiye; ^2^Department of Child Care and Youth Services, Child Development Program, Health Services Vocational School, Akdeniz University, Antalya, Türkiye

**Keywords:** social skills, problem behaviors, academic self-esteem, teacher-child relationship, preschool

## Abstract

**Introduction:**

Research has shown that children's social skills have effects on their problem behaviors. However, the mechanisms through which social skills contribute to reducing problem behaviors need further clarification.

**Methods:**

This study examined the relationship between preschool children's social skills and problem behaviors, as well as the independent and serial mediating roles of academic self-esteem and teacher-child relationship in this relationship. The study included 382 children aged 3–5 years, randomly selected in Türkiye.

**Results and discussion:**

As a result of the study: (1) there was a significant and negative relationship between problem behaviors and social skills, academic self-esteem and teacher-child relationship; (2) the mediating role of academic self-esteem and teacher-child relationship in the relationship between social skills and problem behaviors was supported; and (3) the serial mediating role of academic self-esteem and teacher-child relationship in the effect of social skills on problem behaviors, respectively, was found. These findings indicated that children's social skills may help to increase their academic self-esteem, improve the teacher-child relationship, and thus reduce their problem behaviors. Therefore, these results have important implications for designing interventions to increase preschool children's social skills, academic self-esteem, and teacher-child relationship, as well as to prevent the early development of problem behaviors.

## Introduction

A growing body of research has focused on elucidating the mechanisms underlying children's PB (Problem behaviors) (e.g., Baardstu et al., 2022b; Choi et al., 2022; Rademacher et al., 2022; Tan et al., 2023). PB are evaluated in two categories: (1) externalizing behaviors, which consist of criminal and aggressive behaviors such as fighting, bullying, kicking, biting, hitting, teasing, and threatening; and (2) internalizing behaviors, which reflect internal states such as anxiety, depression, and withdrawal (Baardstu et al., 2022b; Berry and O'Connor, 2010; Walker, 2006). These PB usually emerge early in life and may lead to permanent PB if necessary interventions are not made. For example, in a study examining the effect of early behavioral disorders on academic achievement in high school, it was determined that children's attention, internalizing behavior and externalizing problems at the age of 6 significantly predicted their math and reading achievement at the age of 17 (Breslau et al., 2009). In addition, many studies have shown that children's PB are associated with school readiness (Yong et al., 2022), school success (McDermott et al., 2022), reading difficulties (Trzesniewski et al., 2006), low level of self-regulation (Rademacher et al., 2022), the quality of the TCR (Wang et al., 2022), academic achievement (Kremer et al., 2016), and later delinquency (Hay and Pawlby, 2003), substance abuse, financial, work, and mental health problems (Moffitt et al., 2002). However, limited research has been conducted on the mechanisms underlying this relationship. Examining the development of such problems before PB become persistent in the early years can help design effective interventions targeting preschool children who are most likely to benefit. The purpose of this study was to examine the relationship between preschool children's social skills (SS) and PB, as well as the potential independent and serial mediating roles of academic self-esteem (ASE) and teacher-child relationship (TCR) in this relationship.

### The effect of SS on PB

SS, which include specific learned behaviors, consist of both initiating communication and responding appropriately, and require interaction with others (Little et al., 2017). Children who are considered social in this respect are defined as friendly, responsible and socially capable individuals (Basharpoor et al., 2022). Because of the critical role that SS play in children's socially adaptive behaviors, they are often studied in relation to PB (e.g., Elliott et al., 2019; Montroy et al., 2014). Existing evidence has also revealed that different negative outcomes such as PB (Maag, 2005), guilt, depression, social withdrawal (Cook et al., 2008), school dropout (Elksnin and Elksnin, 1998), academic failure (Malecki and Elliot, 2002) are risk factors for children's SS deficits. SS deficits include not making eye contact consistently when talking to someone, interrupting others, aggression, and other maladaptive behaviors (Little et al., 2017) and can have detrimental effects on a child's current and future level of functioning (Danielson and Phelps, 2003). For example, it has been found that children with SS deficits have higher rates of juvenile delinquency (Elksnin and Elksnin, 1998) and are more likely to have low frustration tolerance, emotional dysregulation (Beaumont et al., 2017), low academic performance (Cook et al., 2008), and there is a strong negative correlation between PB and social competence (Hukkelberg et al., 2019). In addition, previous research has indicated that both SS and PB dimensions represent not only interrelated but also independent components of social competence (Heyman et al., 2018; Montroy et al., 2014). Therefore, early childhood is an important focus for developing children's SS and reducing PB.

### Potentially mediating role of ASE

One possible mechanism underlying the relationship between SS and PB is children's ASE. ASE, which involves children's understanding of their academic skills from both their own and others' perspectives, is a non-cognitive factor (Cámara-Martínez et al., 2023; Giofr et al., 2017; Warash and Markstrom, 2001). Indeed, previous research has examined the links between ASE and academic self-efficacy and academic achievement (Ahmadi, 2020), school adjustment (Basharpoor et al., 2022), academic self-concept (Basith, 2021), intelligence, working memory, academic achievement (Giofr et al., 2017) and SS (Kiliç et al., 2021). The results showed that there is a significant relationship between ASE and academic self-concept (Basith, 2021), and ASE has an indirect effect on academic achievement mediated by intelligence (Giofr et al., 2017). In addition, it has shown that school adjustment is positively related to academic self-concept, and that children's high social acceptability can affect school adjustment both directly and indirectly through school engagement and academic self-concept (Basharpoor et al., 2022). It was also found that low self-esteem is associated with children's use of maladaptive achievement strategies, which in turn is associated with their maladjustment at school, internalizing and externalizing PB (Aunola et al., 2000). Theoretically, Hinshaw (1992) proposes a model of reciprocal relationships between problem behaviors and academic achievement. According to this model, academic achievement and problem behaviors occur simultaneously and each area leads to the other. Moreover, the problems of children with academic underachievement are not limited to the academic field, self-esteem deficits, problems related to SS, and conflict in TCR are also commonly seen (e.g., Ahmadi, 2020; Basharpoor et al., 2022; Basith, 2021; Giofr et al., 2017). Given the strong associations of ASE with both SS and PB, as mentioned above, the current study tested the mediating role of ASE in the relationship between the two variables.

### Potentially the mediating role of TCR

Many influential theoretical frameworks such as Bronfenbrenner's (2005) ecological systems theory, Dodge's social information processing theory Dodge and Rabiner (2004) and Bowlby's (1946) attachment theory, Vygotsky's (1981) sociocultural theory state that children's social, emotional and academic development is directly related to the quality of their interactions with parents, teachers and peers in their environment. In this respect, the TCR in the preschool period is very important for children's social, emotional and academic development. Existing evidence has also revealed that TCR is related to factors such as children's academic achievement, SS and social development, and PB (Bratsch-Hines et al., 2023; Buyse et al., 2008; Chen et al., 2021; Doumen et al., 2008; Glüer and Gregoriadis, 2017; Lei et al., 2023; Palermo et al., 2007; Zhu et al., 2023). In this context, research shows that the quality of TCR shapes children's development in different aspects, including the development of PB (e.g., Buyse et al., 2008; Doumen et al., 2008; Zhu et al., 2023).

In shaping the TCR, a child's externalizing behaviors, such as aggression, can promote maladaptive interactions and conflict with the teacher, which can mutually reinforce the child's PB over time. The persistence of PB may confirm the teacher's negative expectations and thus perpetuate the teacher's negative interactions with the child (Doumen et al., 2008). For example, Paes et al. (2023) found that conflict in the TCR in preschool is associated with poorer SS, particularly lower assertion and lower engagement. Longitudinal studies have also shown that this negative and conflictual relationship has negative effects on children in the long run, such as increased aggressive behaviors, negative school attitudes, less positive school attendance, and lower academic achievement (Bratsch-Hines et al., 2023; Doumen et al., 2008, 2011; Li et al., 2022). For example, Li et al. (2022) found that teacher-child conflict from kindergarten to first grade was associated with academic skills in primary school. In contrast, positive TCR allow children to develop and use effective SS to negotiate and overcome difficulties (Rudasill and Rimm-Kaufman, 2009). For example, Zhu et al. (2023) found that positive TCR buffered the relationship between social avoidance and peer exclusion in preschool children. It is also stated that teachers' use of strategies that support the development of emotional intelligence plays a critical role in reducing children's problem behaviors by increasing their ASE levels (Chaidi and Drigas, 2022). Therefore, it seems reasonable to consider the mediating role of TCR in the link between preschool children's SS and PB.

### The serial mediation role of ASE and TCR in the relationship between SS and PB

As discussed above, both ASE and TCR can independently mediate the relationship between SS and PB. However, how these two mediators work together in this relationship has yet to be explored. According to Hayes (2017), when a mediation model includes more than one mediator, these mediators can play a serial mediation role if they are interrelated. Existing studies have proven the close relationship between ASE and TCR (Chen et al., 2021; Cui et al., 2020; Doumen et al., 2011; Lei et al., 2023; van Aalst et al., 2021). For example, Cui et al. (2020) showed that the TCR helps to increase children's self-confidence, which has a positive relationship with academic achievement. Therefore, it is possible that ASE and TCR not only play an independent mediating role in the relationship between SS and PB, but also relate to each other and also play a serial mediating role in this relationship.

Consistent with the ideas expressed by previous studies (Baardstu et al., 2022a; Berry and O'Connor, 2010; Hinshaw, 1992; Kiliç et al., 2021; Wang et al., 2022), the theoretical model in the current study was constructed by taking into account that as children's PB increase, their SS, TCR and ASE are negatively affected by this situation. In addition, in the model proposed in the current study, it is assumed that ASE and TCR have independent and serial mediator roles in the relationship between preschool children's SS and PB. As shown in the literature, children's ASE (e.g., Arnold et al., 2012; Lane et al., 2005) and TCR (e.g., Wang et al., 2022) are important predictors of both SS and PB. Moreover, in line with the propositions of the Bioecological systems perspective (Bronfenbrenner, 2005), TCR and ASE will be associated with children's SS and PB. Therefore, it is reasonable to expect that preschool children's SS and PB may have different effects on ASE and TCR.

### The current study

**Figure 1:**
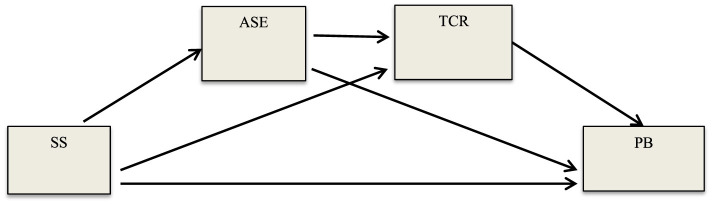
The estimation model suggested in the research.

Although researchers have separately conducted extensive research to understand the relationships between preschool children's SS, PB, TCR, and ASE variables (e.g., Bratsch-Hines et al., 2023; Berry and O'Connor, 2010; Choi et al., 2022; Doumen et al., 2008; Rudasill and Rimm-Kaufman, 2009; Zhu et al., 2023), to the best of our knowledge, no study has tested the mediating roles (Hayes, 2017) of the underlying causes of these variables. Therefore, analyzing the different mechanisms that play a role in these relationships will further help to understand the factors that protect children from or put them at risk of these serious problem situations. In addition, this study contributes to the existing literature by including different assessments of both SS and PB than most previous studies. In this respect, the current study was conducted to examine the relationship between preschool children's SS and PB, as well as the independent and serial mediating roles of ASE and TCR in this relationship (the mediation model in Figure 1 was tested). In the current study, the following hypotheses were developed under the guidance of previous studies.

H_1_: There is a significant relationship between SS, PB, TCR and ASE.

H_2_: The effect of SS on PB is significant.

H_3_: The mediating role of ASE in the effect of SS on PB is significant.

H_4_: The mediating role of TCR in the effect of SS on PB is significant.

H_5_: The serial mediation role of ASE and TCR is significant in the effect of SS on PB, respectively.

## Methods

### Participants

The study included 382 children aged 3–5 years and their teachers (*n* = 40) randomly selected from 3 kindergartens and 12 preschools in Bayburt, Türkiye. Demographic information about the children and teachers was obtained through the consent form for participation in the study sent to the teachers along with the measurement tools and teacher responses. Accordingly, 197 (51.6%) of the children in the study were girls and 185 (48.4%) were boys. In terms of age, 4 (1%) were 3 years old, 37 (9.7%) were 4 years old and 341 (89.3%) were 5 years old. All of the teachers (*n* = 40) who assessed children's SS, PB, ASE and TCR were female and their ages ranged between 22 and 42 years (M_age_ = 30.7, SD = 6.1). In terms of the length of professional service of the teachers, 2 (5%) had been working in preschool education institutions for 6–11 months, 16 (40%) for 1–5 years, 13 (32.5%) for 6–10 years and 9 (22.5%) for more than 10 years.

### Measures

The data were obtained using the Social Skills Improvement System-Rating Scales to assess children's social skills and PB, the Academic Self-Esteem Scale to assess their ASE, and the Student-Teacher Relationship Scale-Short Form for the TCR.

#### Social skills improvement system-rating scales (SSIS-RS) (preschool ages 3–5)

The SSIS-RS (Gresham and Elliott, 2008) was used to capture data on a wide range of students' social behaviors and academic functioning. The SSIS-RS includes two subscales: the SS subscale, and the PB subscale. The preschool version of SSIS-RS included parents' and teachers' ratings. In the current study, children's SS and PB were all assessed by their teachers. The SS subscale consists of 46 items and seven sub-domains: communication, cooperation, assertion, responsibility, empathy, engagement, and self-control. The PB subscale consists of 30 items and five sub-domains: externalizing, bullying, hyperactivity/inattention, internalizing and autism spectrum. The items and scoring of the SS and PB scales use a 4-point frequency scale with Never = 0, Rarely = 1, Often = 2, and Almost Always = 3 as scale anchor points. The SSIS-RS included the following items: “Follows your directions,” “Takes turns in conversations,” “Follows classroom rules,” and “Acts without thinking” (Gresham and Elliott, 2008).

The internal consistency coefficients of the SSIS-RS were as follows: communication 0.84, cooperation 0.89, assertion 0.83, responsibility 0.90, empathy 0.86, engagement 0.84 and self-control 0.86, externalization 0.93, bullying 0.74, hyperactivity/inattention 0.88, internalization 0.78 and autism spectrum 0.84 (Gresham and Elliott, 2008). It was concluded that the internal consistency coefficients of The SSIS-RS teacher form for 3–5-year-old children in Türkiye ranged between 0.70 and 0.86 and that it is a valid and reliable tool for assessing children's SS and PB (Tutkun and Dinçer, 2019).

#### Academic self-esteem scale

The Academic Self-Esteem Scale (ASES) was developed by Cevher and Buluş (2006) to measure the ASE of preschool children. In the process of developing the scale, the Behavioral ASES developed by Coopersmith and Gilberts (1982) and the Assessment Inventory developed by Hamachek (1995) were used. The scale includes the following statements: “He puts forward new ideas in the classroom. He/she is spontaneously motivated for school work.” The range of points that can be obtained from the 5-point Likert-type ASES is between 22 and 110. The higher the score on the scale, the higher the level of ASE of the children, and the lower the score, the lower the level of ASE. In the internal consistency reliability analysis, Cronbach Alpha (α) of the scale was found to be 0.95.

#### Student-teacher relationship scale-short form

The Student-Teacher Relationship Scale (STRS) (Pianta and Nimetz, 1991) assesses teachers' levels of closeness and conflict with each child in their classroom. The closeness subscale included 8 items (e.g., This child values his/her relationship with me), and the conflict subscale included 7 items (e.g., This child easily becomes angry at me). For each item, the teacher rates the extent to which the statement reflects the relationship with each child on a scale of 1 (Definitely does not apply) to 5 (Definitely applies). The minimum score for the conflict subscale is 8 and the maximum score is 40. The minimum score on the closeness subscale is 7 and the maximum score is 35. Higher scores indicate higher closeness and lower conflict. The internal consistency coefficient of the scale is between 0.86 and 0.89. The internal consistency coefficients of the STRS form for children in Türkiye were 0.84 for the conflict subscale and 0.76 for the closeness subscale 0.76 for the conflict subscale and 0.84 for the closeness subscale, and it was concluded that it is a valid and reliable tool for assessing the TCR (Asi and Karabay, 2018).

### Procedures

Data were collected by the author. Prior to the study, permission was obtained from the schools and teachers. First of all, a document explaining the general purpose of the study and an information form were given to all teachers in the participating classrooms, and all teachers gave signed consent for participation. Teachers were then given the SSIS-RS to assess children's SS and PB, the ASES to assess ASE, and the STRS-Short Form for TCR. Teachers completed the forms for the children in their classrooms within a 1-week period. No remuneration was offered to the teachers who took part in the study.

Correlations were calculated to determine the relationships between variables. Then, the direct and indirect effects of the independent variable on the dependent variable were investigated. The macro program PROCESS v3.5 (Hayes, 2017) was used to test the hypothesized mediation models and the Sobel test was used to confirm the consistency of the results. The Sobel test is another test that measures whether the mediating variable is statistically significant (Sobel, 1982). SPSS PROCESS macro based on Bootstrap sampling was used to determine the significance of mediating variables in this relationship. During this analysis, bias corrected bootstrap confidence intervals (a bootstrap sample of 5,000 was specified) were calculated at 99% confidence level.

## Results

### Descriptive statistics and correlation analysis

In line with the first hypothesis of the study, the relationships between preschool children's SS, PB, relationships with teachers and ASE were examined (see Table 1). In the study, a negative and statistically significant relationship was determined between preschool children's SS and PB (*r* = −0.58, *p* < 0.01). A positive significant relationship was found between children's SS and their ASE (*r* = 0.71, *p* < 0.01) and their relationships with their teachers (*r* = 0.47, *p* < 0.01). There was also a significant and negative relationship between children's PB and their ASE (*r* = −0.56, *p* < 0.01) and their relationships with their teachers (*r* = −0.62, *p* < 0.01). There was a positive and significant relationship between children's relationships with their teachers and their ASE (*r* = 0.53, *p* < 0.01). In this context, it was concluded that there was a significant relationship between all variables.

**Table 1:** Descriptive statistics and correlations among study variables.

		**Correlations**				**Descriptive statistics**		
		**1**	**2**	**3**	**4**	***n***	* $\bar{X}$ *	***S***
1	SS	1				382	1.96	0.48
2	PB	−0.58^*^	1			382	0.52	0.40
3	ASE	0.71^*^	−0.56^*^	1		382	3.72	0.76
4	TCR	0.47^*^	−0.62^*^	0.53^*^	1	382	4.13	0.55

^*^p < 0.01.

### The effect of SS on PB

**Figure 2:**
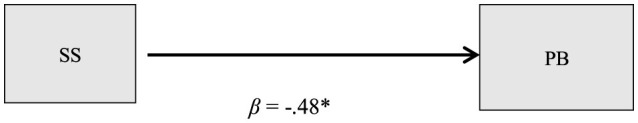
The effect of SS on PB. *p < 0.001.

In line with the second hypothesis of the study, the effect of preschool children's SS on PB was examined (see Figure 2). As a result of the analysis, it was determined that the total effect of SS of preschool children on PB was significant (β = −0.48, SE = 0.03 *p* < 0.001, CI = [−0.57, −0.39]). In other words, SS were found to be a significant predictor of PB. Accordingly, the second hypothesis of the study was accepted.

### The mediating role of ASE in the effect of SS on PB

**Figure 3:**
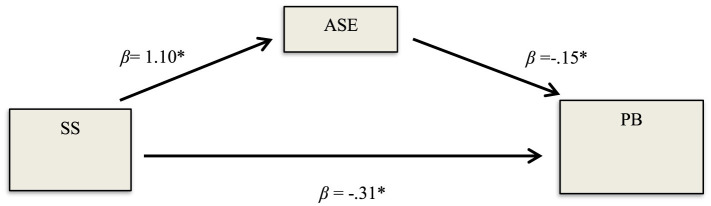
The mediating role of ASE in the effect of SS on PB. *p < 0.001.

In line with the third hypothesis of the study, the mediating role of ASE in the effect of preschool children's SS on their PB was examined (see Table 2). In the analyses, it was determined that the effect of preschool children's SS on ASE, which was determined as the mediating variable, was significant (β = 1.10, SE = 0.05 *p* < 0.001). In addition, the effect of children's ASE on PB was also found to be significant (β = −0.15, SE = 0.03 *p* < 0.001). When ASE, which was determined as a mediating variable, was controlled, the effect of SS on PB decreased (β = −0.31, SE = 0.04 *p* < 0.001). The indirect effect of children's SS on PB through ASE was significant (β = −0.17 SE = 0.03 CI [−0.25, −0.08]). At the same time, the fully standardized indirect effect size of children's SS on PB through ASE (*K*^2^ = −0.20) was significant and high (see Figure 3). The results obtained from the Sobel test also proved that the mediating role of ASE in the effect of SS on PB was significant (Sobel *z* = −4.94, *p* < 0.001). In line with the findings, the third hypothesis of the study was accepted.

**Table 2:** The mediating role of ASE in the effect of SS on PB.

	**Outcome variables**			
	→**M (ASE)**		→**Y (PB)**	
**Predictor variables**	β	***SE***	β	***SE***
Constant	1.54^*^	0.11	1.71^*^	0.08
X (SS)	1.10^*^	0.05	−0.31^*^	0.04
M (ASE)			−0.15^*^	0.03
*R^2^*	0.50		0.38	
*F*	376.69^*^		116.59^*^	
Bootstrap	SS → ASE → PB			
Indirect effect	*K*^2^ *=* −0.20 β = −0.17 SE = 0.03 99% CI [% 0.25, −0.08]			

^*^p < 0.001; *n* = 382; CI, Bootstrap lower and upper confidence interval; SE, Standard error; *K*^2^, Fully standardized effect size. Bootstrap resampling = 5000. Non-standardized beta coefficients (β) were reported.

### The mediating role of TCR in the effect of SS on PB

**Figure 4:**
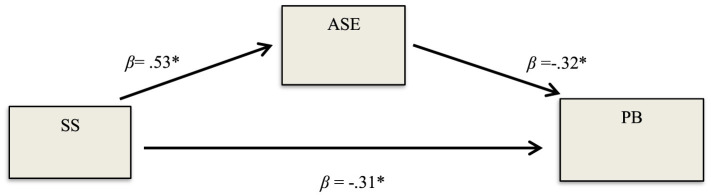
The mediating role of TCR in the effect of SS on PB. *p < 0.001.

In line with the fourth hypothesis of the study, the mediating role of preschool children's SS on their PB was examined (see Table 3). The results showed that the effect of preschool children's SS on their relationships with their teachers was significant (β = 0.53, SE = 0.05 *p* < 0.001). In addition, the effect of children's relationship with their teacher on PB was also significant (β = −0.32, SE = 0.03 *p* < 0.001). When the child's relationship with the teacher, which was determined as the mediating variable, was controlled, the effect of SS on PB (direct effect) decreased (β = −0.31, SE = 0.03 *p* < 0.001). The indirect effect of children's SS on PB through their relationship with their teachers was significant (β = −0.17 SE = 0.02 CI [−0.25, −0.10]). At the same time, the fully standardized indirect effect size of children's SS on PB through their relationships with their teachers (*K*^2^ = −0.20) was significant and high (see Figure 4). The result obtained from the Sobel test also proved the mediating role of the TCR in the effect of SS on PB (Sobel *z* = −7.44, *p* < 0.001). Accordingly, the fourth hypothesis of the study was accepted.

**Table 3:** The mediating role of TCR in the effect of SS on PB.

	**Outcome variables**			
	→**M (TCR)**		→**Y (PB)**	
**Predictor variables**	β	***SE***	β	***SE***
Constant	3.08^*^	0.10	2.47^*^	0.11
X (SS)	0.53^*^	0.05	−0.31^*^	0.03
M (TCR)			−0.32^*^	0.03
*R^2^*	0.22		0.49	
*F*	108.09^*^		182.68^*^	
Bootstrap	SS → TCR → PB			
Indirect effect	*K^2^ =* −0.20 β = −0.17 SE = 0.02 99% CI [−0.25, −0.10]			

^*^p < 0.001; *n* = 382; CI, Bootstrap lower and upper confidence interval; SE, Standard error; *K*^2^, Fully standardized effect size. Bootstrap resampling = 5000. Non-standardized beta coefficients (β) were reported.

### The serial mediation role of ASE and TCR in the effect of SS on PB, respectively

**Figure 5:**
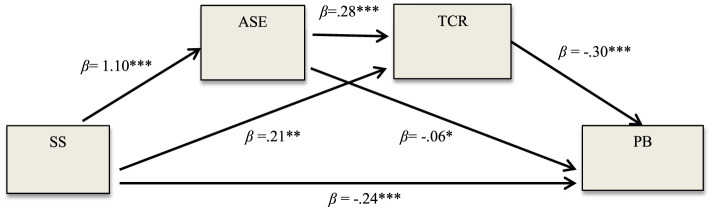
The serial mediation role of ASE and TCR in the effect of SS on PB, respectively. *p < 0.05, **p < 0.01, and ***p < 0.001.

In line with the fifth hypothesis of the study, the serial mediation role of ASE and relationships with teachers on the effect of preschool children's SS on their PB was examined (see Table 4). The results showed that the effect of children's SS on ASE, which was identified as the first mediating variable, was significant (β = 1.10, SE = 0.05 *p* < 0.001). It was also determined that the effect of children's ASE on the child's relationship with the teacher, which was determined as the second mediating variable, was also significant (β = 0.28, SE = 0.04 *p* < 0.001). It was also found that children's relationship with their teachers had a significant effect on PB (β = −0.30 SE = 0.03 *p* < 0.001). It was concluded that ASE and relationship with the teacher played a serial mediating role in the effect of children's SS on PB, respectively (β = −0.09 SE = 0.01 CI = [−0.15, −0.05]). At the same time, the full standardized indirect effect size (*K*^2^ = −0.11) of preschool children's SS on PB through serial mediation of their ASE and their relationship with their teachers, respectively, was determined to be significant and moderate (see Figure 5). Moreover, the result obtained from the Sobel test proved the serial mediation role of ASE and TCR, respectively, in the effect of SS on PB (Sobel *z* = −5.49, *p* < 0.001). In line with the findings, the fifth hypothesis of the study was accepted.

**Table 4:** The serial mediation role of ASE and TCR in the effect of SS on PB, respectively.

	**Outcome variables**					
	→**M**_1_ **(ASE)**		→**M**_2_ **(TCR)**		→**Y (PB)**	
**Predictor variables**	β	***SE***	β	***SE***	β	***SE***
Constant	1.54^*^	0.11	2.63^*^	0.11	2.50^*^	0.11
X (SS)	1.10^*^	0.05			−0.24^*^	0.04
M_1_ (ASE)			0.28^*^	0.04		
M_2_ (TCR)					−0.30^*^	0.03
*R^2^*	0.49		0.30		0.49	
F	376.69^*^		81.77^*^		125.10^*^	
Bootstrap	SS → ASE → TCR → PB					
Indirect effect	*K*^2^ *=* −0.11 β = −0.09 SE = 0.01 99% CI [−0.15, −0.05]					

^*^p < 0.001; *n* = 382; CI, Bootstrap lower and upper confidence interval; SE, Standard error; *K*^2^, Fully standardized effect size. Bootstrap resampling = 5000. Non-standardized beta coefficients (β) were reported.

## Discussion

The current study examined the relationship between SS and PB of preschool children, as well as the independent and serial mediator roles of ASE and TCR in this relationship. As a result of the study: (1) there was a significant and negative relationship between PB and SS, ASE and TCR; (2) SS were found to be a significant predictor of PB; (3) the mediating role of ASE and TCR in the relationship between SS and PB was supported; and (4) the serial mediating role of ASE and TCR in the effect of SS on PB, respectively, was found.

As a result of the study, negative relationships were found between preschool children's PB and their SS, ASE and TCR. In addition, SS were found to be a significant predictor of PB. These results supported the first and second hypotheses of the study. Accordingly, as children's SS, ASE and TCR increased, their PB decreased. Similarly, children with high levels of SS, ASE, and TCR exhibit fewer PB and contribute to the significant evidence that PB have a negative relationship with SS, ASE, and TCR (Elliott et al., 2019; Heyman et al., 2018; Maag, 2005; Kremer et al., 2016). This relationship can be justified as follows: As children's relationships with their teachers improve, their ASE and SS increase, their PB decrease: PB make it difficult for children to establish healthy relationships with their environment and adapt to the social environment they are in. This situation negatively affects his/her relationships with teachers and peers. As a matter of fact, children's exhibiting PB due to lack of SS is likely to create a negative belief on their ASE. ASE, which includes children's feelings about their academic success and ability (Warash and Markstrom, 2001) and is considered as a non-cognitive factor (Giofr et al., 2017), is also affected by this negative relationship and may prevent children from participating more actively in learning processes and being successful. For example, Walker (2006) found that ASE and aggressive behaviors of preschool children are interrelated. Early PB predicted future academic success and difficulties (Breslau et al., 2009; Miner and Clarke-Stewart, 2008). In this respect, children with PB and a history of academic failure are more likely to develop lower ASE than children with fewer experiences of academic failure and SS.

The independent mediating role of ASE in the relationship between preschool children's SS and PB was supported in this study. The present results support and extend the evidence that social and academic development are interconnected from early on. Previous studies have shown that kindergarten children's learning achievement is most strongly related to SS (Offer-Boljahn et al., 2022) and that there is a high positive relationship between SS and academic achievement (Okeke et al., 2022). On the other hand, PB such as aggression, introversion, bullying, hyperactivity/inattention and internalizing disorders negatively affect children (Adetunji et al., 2022), prevent young children from succeeding in the classroom (McDermott et al., 2022), that poor SS are associated with short-, medium-, and long-term adjustment difficulties (Gresham, 2016), that this has a lasting effect in the future (Kremer et al., 2016; Moffitt et al., 2002), that consistently low SS increase the risk of poor school performance, while consistently high SS increase the chances of good school performance in primary school (Frogner et al., 2022). These results suggest that increasing children's SS and ASE at an early stage may be helpful, especially in terms of preventing or reducing the emergence of PB.

The current study also found that the TCR may be an important mediator of the relationship between SS and PB. This finding supported Bronfenbrenner's (2005) ecological systems theory, which states that children develop by being influenced by their interactions with individuals in their immediate environment. This finding also confirms that TCR play a role in helping children acquire the social and academic skills necessary for success in school and in reducing PB. In addition, previous research has shown that children's aggressive behaviors lead to an increase in teacher-child conflict, which in turn leads to an increase in aggressive behaviors (Doumen et al., 2008); children's PB negatively affect teachers' closeness with children, while children's SS positively affect teachers' closeness with children (Glüer and Gregoriadis, 2017); externalizing and internalizing problems are negatively associated with high teacher-child closeness (Wang et al., 2022); that teachers have more conflictual relationships with children with behavior problems (Buyse et al., 2008); and that TCR is a moderator of the relationships between classroom interactions and children's classroom behavior (Lippard et al., 2018). The current study also found that the TCR may be an important mediator of the relationship between SS and PB. SS can reduce children's PB, which can help children build better relationships with their teachers within the classroom atmosphere, thus improving the TCR.

The serial mediating role of ASE and TCR in the relationship between preschool children's SS and PB is also significant in this study. Such a finding, based on the independent mediating role of TCR, revealed that ASE is closely related to SS and PB simultaneously, thus, it forms a serial mediation pathway between SS and PB together with TCR. This finding also extends and supports previous research using structural equation modeling (Ahmadi, 2020; Baardstu et al., 2022b; Chen et al., 2021; Cui et al., 2020; Paes et al., 2023; Palermo et al., 2007; van Aalst et al., 2021; Wu et al., 2022; Zhu et al., 2023). For example, Paes et al. (2023) found that teacher-child conflict was associated with poorer SS and PB in children; Zhu et al. (2023) found that teacher-child conflict exacerbated the relationships between social avoidance and peer exclusion and anxious-fearful behavior; and self-confidence in learning mediated significant relationships between TCR and students' academic achievement (Cui et al., 2020). In addition, it was found that teacher-child closeness mediated the relationship between school engagement and shyness (Wu et al., 2022), social avoidance and peer exclusion (Zhu et al., 2023), children's behavior and peer exclusion mediated the relationship between TCR and academic readiness (Palermo et al., 2007), and ASE was directly related to academic self-efficacy and indirectly related to academic achievement (Ahmadi, 2020). In the light of all these results, it is important to consider mechanisms such as ASE and TCR that affect the relationship between preschool children's SS and PB when designing educational interventions.

## Conclusions, limitations and implications

In conclusion, this study examined the relationship between preschool children's SS and PB, as well as the independent and serial mediating roles of ASE and TCR in this relationship. The results showed that preschool children's ASE and their relationship with their teachers were directly and indirectly related to their SS and PB through the independent and serial mediator role.

The current study has some limitations. First, although the current study revealed possible contemporaneous relationships between all study variables, it could not demonstrate long-term, causal relationships between them without a longitudinal study. Second, the data for each study variable in the current study was based on teacher ratings. Future studies should try to include multiple sources of information, especially parents and/or teachers, and direct observations of children. This is because existing evidence has revealed inconsistencies in parent and teacher ratings (Gresham et al., 2010; Heyman et al., 2018; Wang et al., 2022). Third, the current study did not address other variables such as child age, gender, temperament, or parental attitudes in ASE, TCR, SS, and PB. How and in what ways such variables affect children's ASE, TCR, SS and PB can be further investigated. On the other hand, cultural differences can affect individuals' behaviors, socialization processes, and the way they perceive the world in various ways. Given the variety of indirect or direct effects of culture (Vaughn et al., 2014), this study focuses on preschool children growing up in Turkish culture. However, future studies may examine children from different cultural contexts to assess the effects of culture on ASE, TCR, SS and PB from a broader perspective. Indeed, existing evidence from previous studies has also revealed that there are cultural differences in child and teacher contexts (e.g., Chen and Roorda, 2025; Xu et al., 2023). In this context, conducting similar studies with children in different cultural contexts is important for generalizability.

The current research theoretically contributed to the understanding of the mechanisms underlying the impact of SS and PB, such as children's ASE and TCR, and identified a valuable direction for future studies to analyze their causes. Practically, the predictive models revealed in the current study will be useful for educators and other professionals to successfully develop interventions and programs based on available evidence to improve children's SS, ASE, and relationships with their teachers and to reduce PB. At the same time, understanding the determinants of TCR can inform teacher preparation and professional development and increase teachers' awareness of the characteristics that can influence children's development and achievement. In addition, by having a deeper understanding of how to prevent children's PB and improve their relationships with children, teachers can promote and develop high-quality relationships with more children.

## Data Availability

The original contributions presented in the study are included in the article/supplementary material, further inquiries can be directed to the corresponding author.
